# A Deep Learning Model for the Automatic Recognition of Aplastic Anemia, Myelodysplastic Syndromes, and Acute Myeloid Leukemia Based on Bone Marrow Smear

**DOI:** 10.3389/fonc.2022.844978

**Published:** 2022-04-14

**Authors:** Meifang Wang, Chunxia Dong, Yan Gao, Jianlan Li, Mengru Han, Lijun Wang

**Affiliations:** Department of Hematology, The Second Hospital of Shanxi Medical University, Taiyuan, China

**Keywords:** aplastic anemia, myelodysplastic syndromes, myeloid leukemia, identification model, convolutional neural networks

## Abstract

**Aim:**

Bone marrow biopsy is essential and necessary for the diagnosis of patients with aplastic anemia (AA), myelodysplastic syndromes (MDS), and acute myeloid leukemia (AML). However, the convolutional neural networks (CNN) model that automatically distinguished AA, MDS, and AML based on bone marrow smears has not been reported.

**Methods:**

Image-net pretrained model of CNN was used to construct the recognition model. Data extracted from the American Society of Hematology (ASH) Image Bank were utilized to develop the model and data extracted from the clinic were used for external validation. The model had two output layers: whether the patient was MDS (two-classification) and which of AA, MDS, and AML the patient was (three-classification). Different outcome weights (two-classification/three-classification = 5:5, 2:8, 1:9) and epochs (30, 50, 200) were used to select the optimal model. The model performance was evaluated by the Accuracy-Loss curves and calculating the area under the curve (AUC), accuracy, sensitivity, specificity, positive predictive value (PPV), and negative predictive value (NPV).

**Results:**

A total of 115 bone marrow smears from the ASH Image Bank and 432 bone marrow smears from the clinic were included in this study. The results of Accuracy-Loss curves showed that the best model training effect was observed in the model with the outcome weight and epoch of 1:9 and 200. Similarly, this model also performed well performances in the two-classification of MDS and the three-classification of AA, MDS, AML. The AUC, accuracy and sensitivity of the MDS two-classification model in the testing set were 0.985 [95% confidence interval (CI), 0.979-0.991], 0.914 (95%CI, 0.895-0.934), and 0.992 (95%CI, 0.980-1.000), respectively. The AUC, accuracy and sensitivity of the AA, MDS, AML three-classification model in the testing set were 0.968 (95%CI, 0.960-0.976), 0.929 (95%CI, 0.916-0.941), and 0.857 (95%CI, 0.828-0.886), respectively.

**Conclusion:**

The image-net pretrained model was able to obtain high accuracy AA, MDS, AML distinction, and may provide clinicians with a convenient tool to distinguish AA, MDS, and AML.

## Introduction

Myelodysplastic syndromes (MDS) are myeloid tumors characterized by clonal proliferation of hematopoietic stem cells, recurrent genetic abnormalities, myelodysplasia, ineffective hematopoiesis, and peripheral blood cell reduction, and progression to acute myeloid leukemia (AML) in one-third of patients (1). Furthermore, aplastic anemia (AA) is also a marrow disease that causes pancytopenia, and approximately 15% to 20% of AA patients progress to MDS or AML (2, 3). Therefore, the correct diagnosis has an important influence on the treatment, control, and prognosis of these diseases. The laboratory diagnosis of MDS depends on morphological changes based on peripheral blood and bone marrow dysplasia, including peripheral blood smears, bone marrow aspiration smears, and bone marrow biopsy (4, 5). Since AA and MDS are all accompanied by significant cytopenia, it is difficult to distinguish the two diseases, and both AA and MDS may progress to AML (6, 7). In addition, there may be differences in the accuracy of manual identification of these diseases. A tool that can assist clinicians in automatically distinguishing AA, MDS, and AML may be needed in clinical practice.

Deep learning is a type of machine learning that uses multiple processing layers to learn data representations with multiple levels of abstraction (8). Deep learning methods use the complete image and associate the entire image with the diagnostic output (9). The processing of images in deep learning usually relies on convolutional neural networks (CNN), which is a neural network that is particularly good at classifying images (10). CNN has an outstanding image classification effect because it can imitate the natural visual processing in the brain and can interpret dense information (10). Therefore, the use of deep learning methods to help clinicians diagnose image information is of great significance. Recently, deep learning has been widely used in the identification and classification of diseases (11–13). In the study on MDS recognition, a recent study developed a deep learning model to distinguish AA and MDS based on peripheral blood indicators (14). Bone marrow biopsy is essential and necessary for the diagnosis of patients with AA, MDS (6). However, studies based on bone marrow smears to identify AA, MDS, and AML has not been reported.

Herein, we aimed to develop and validate a model based on bone marrow smears using deep learning methods to identify whether patients had MDS, and to distinguish AA, MDS, and AML patients.

## Methods

### Data Source and Populations

**Figure 1:**
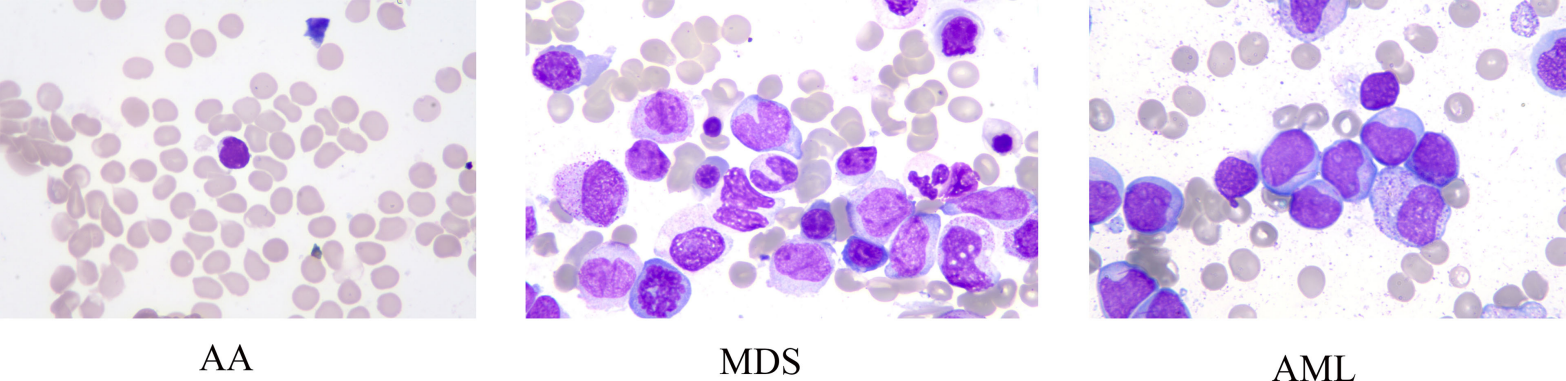
Sample images of aplastic anemia (AA), myelodysplastic syndromes (MDS), and acute myeloid leukemia (AML).

Data of this study were extracted from two different sources: The American Society of Hematology (ASH) Image Bank (15) and The Second Hospital of Shanxi Medical University data from July 2016 to December 2020. The determination of patients with AA, MDS, and AML in the ASH Image Bank was based on the disease category corresponding to the patient’s bone marrow smear in the database. Diagnosis from hospital patients was based on the following criteria. The diagnosis of AA is based on the International Agranulocytosis and Aplastic Anemia criteria (16), that is, the peripheral blood meets at least two of the following three criteria: (1) hemoglobin ≤100 g/L; (2) platelets ≤50×10^9^/L; (3) granulocytes ≤1.5×10^9^/L. The diagnosis of MDS and AML are according to the World Health Organization classification of myeloid neoplasms and acute leukemia criteria (2016 version) (17). In addition, peripheral blood or bone marrow blasts ≥20% is a necessary condition for the diagnosis of AML, but when the patient is confirmed to have clonal and reproducible cytogenetic abnormalities t(8;21)(q22;q22.1), inv(16)(p13.1;q22) or t(16;16)(p13.1;q22), and t(15;17)(q22;q12), even if bone marrow blasts are less than 20%, it should be diagnosed as AML (17). The sample images of AA, MDS, and AML were displayed in **Figure 1**. A total of 115 bone marrow smears were collected from the ASH Image Bank, including 32 were MDS, 26 were AA, and 57 were AML. Similarly, a total of 432 bone marrow smears (MDS, 214; AA, 115; AML, 103) were also extracted from the hospital. Data from the ASH Image Bank were used for model development and internal validation, and data from the hospital were utilized for external validation. The ASH Image Bank is a web-based publicly available image library that provides a comprehensive collection of images related to a wide range of hematologic topics. This study was approved by the Institutional Review Board of The Second Hospital of Shanxi Medical University [approval number: No.2021(162)].

### Data Enhancement

**Figure 2:**
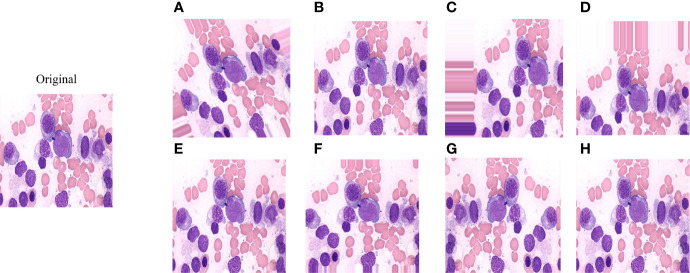
The effect of applying image transformation to the same image sample. **(A)** rotation; **(B)** ZCA whitening; **(C)** width shift; **(D)** height shift; **(E)** shearing; **(F)** zoom; **(G)** horizontal flip; **(H)** vertical flip.

**Figure 3:**
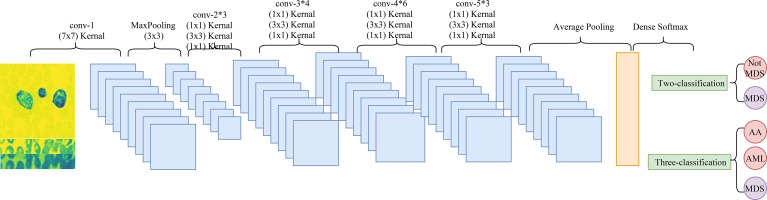
The architectural details of Resnet 50.

**Figure 4:**
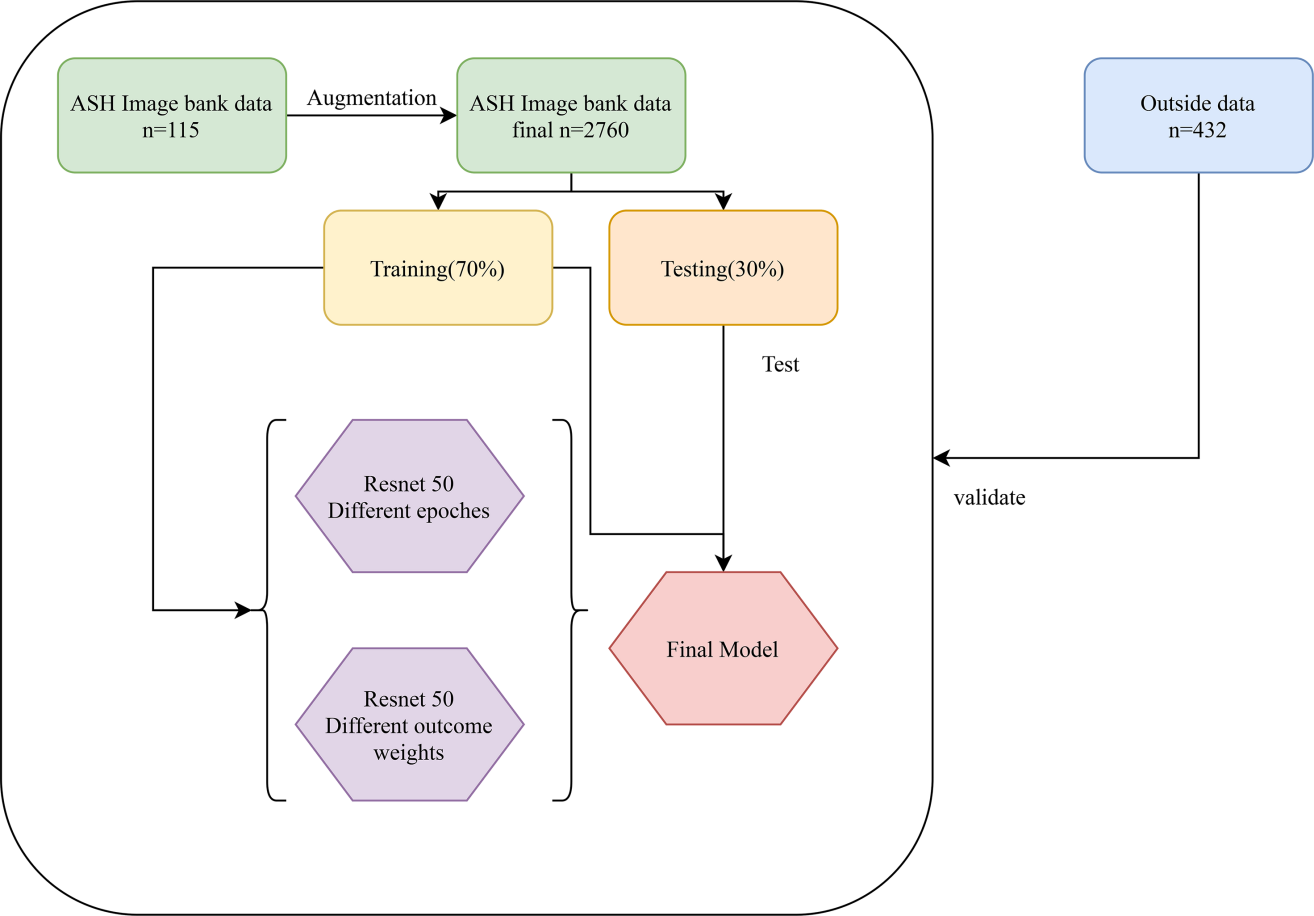
The construction process of the recognition model.

**Figure 5:**
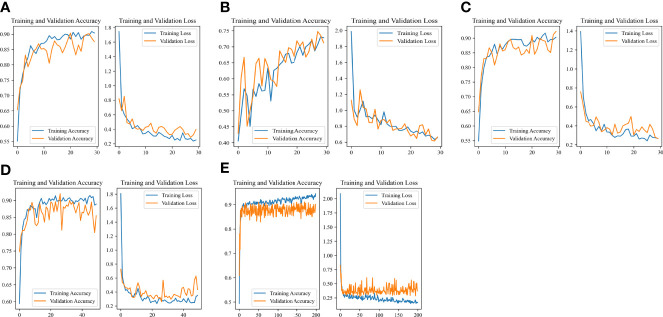
Accuracy-Loss curves of the Resnet 50 Image-Net pretrained model in different outcome weights and epochs. **(A)** 30 epochs and 5:5 outcome weight; **(B)** 30 epochs and 2:8 outcome weight; **(C)** 30 epochs and 1:9 outcome weight; **(D)** 50 epochs and 1:9 outcome weight; **(E)** 200 epochs and 1:9 outcome weight.

**Figure 6:**
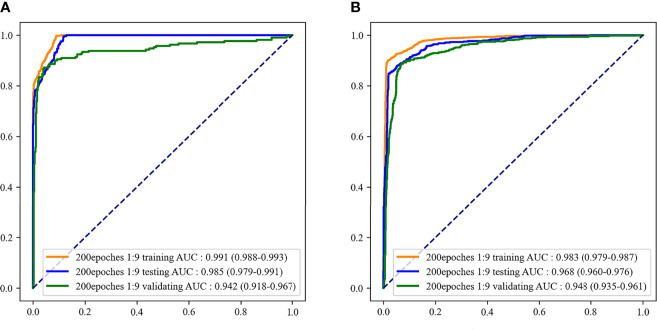
The receiver operator characteristic (ROC) curves of the final recognition models. **(A)** ROC curves of the two-classification model; **(B)** ROC curves of the three-classification model.

Data enhancement methods were widely used to increase the sample size in deep learning algorithms, and reduce the error rate of the model by providing better generalization (18–20). In this study, the number of samples for each disease in the ASH Image Bank is very limited. Eight image transformation methods, including rotation, shift, sheer, flip, were used to obtain different versions of original images, and each original image was increased to three samples using each data enhancement method. After data enhancement, the number of samples in the ASH Image Bank dataset had been expanded by 24 times, from 115 to 2760. The detailed eight image transformations were as follows: (1) rotation (35^○^), the picture was rotated 35 degrees in a random direction (left or right) (**Figure 2A**); (2) ZCA whitening, whitening can be used to reduce redundant information of pictures and preserve important information (**Figure 2B**); (3) width shift (35%), the picture was randomly shifted to the left or right by 40% (**Figure 2C**); (4) height shift (35%), it was obtained by randomly shifting the image to up or down with 35% (**Figure 2D**); (5) shearing (35^○^), it was done by shifting the image counterclockwise by 35 degrees (**Figure 2E**); (6) zoom (35%), the picture was zoomed by 35% to make the appearance of objects in the image closer (**Figure 2F**); (7) horizontal flip, it was obtained by flipping the image up and down (**Figure 2G**); (8) vertical flip, it was obtained by flipping the image left and right (**Figure 2H**).

### Convolutional Neural Network

Convolutional neural network (CNN) is a deep learning model, which includes three main components, convolution layer, pooling layer, and output layer. The convolution layer is used to extract important features in pictures, the pooling layer is utilized to reduce the dimension of features, and the output layer is used for prediction (21). The CNN model used in this study was Resnet 50. **Figure 3** shows the architectural details of Resnet 50.

### Deep Learning Model

The classification of images by deep learning mainly consists of three processes (22): (1) image preprocessing, images used for deep learning are normalized, and all images are resampled to size 256*256; (2) feature extraction and training, images of different diseases may have their features, and the features of images can be extracted and learned through CNN, which is also known as model training; (3) classification, after the model has learned features, the detected objects are classified by appropriate classification techniques that compare the image pattern with the target pattern. Although deep learning can classify images, it is unknown which features deep learning extracts in a single image. The Image-Net pretrained model of the Resnet 50 was used to construct the recognition model. The optimizer of the Resent 50 model was Adams, the loss function was categorical cross-entropy, and the output layer had two layers. Better model parameters are obtained by adjusting the number of times the training set is learned in the model (epochs) and the weight of the two output layers. The two output layers were: (1) whether the patient was MDS (two-classification output layer); (2) which of AA, AML, and MDS the patient was (three-classification output layer). In addition, the two output layers were given different weights during the model building process. The detailed construction processes of the recognition model were as follows: (1) data of the ASH Image Bank were randomly divided into the training set and the testing set with a ratio of 7:3; (2) the weight of the two output layers (two-classifications/three-classifications) was selected as 2:8, and three different epochs (30, 50, and 200) were adopted to assess the impact of different epochs on the recognition models; (3) three different weights (5:5, 2:8, and 1:9) of the two output layers were used to evaluate the influence of different outcome weights on the recognition models; (4) the model performance was evaluated by the Accuracy-Loss curves and calculating the area under the curve (AUC), accuracy, sensitivity, specificity, positive predictive value (PPV), and negative predictive value (NPV). The detailed construction process of the model was shown in **Figure 4**.

### Statistical Analysis

The OpenCV and Keras libraries of Python were used for image enhancement. The construction of the Image-Net pretrained model was performed by the Python 3.8 software.

## Results

### Accuracy-Loss Curves of the Model in Different Outcome Weights and Epochs

The Accuracy-Loss curves of the Resnet 50 Image-Net pretrained model in different outcome weights and epochs were shown in **Figure 5**. For models with different outcome weights, the Accuracy-Loss curves of the model demonstrated that when the outcome weights of the model were 5:5 and 2:8, the model training effect raised slowly, and when the outcome weight of the model was 1:9, the model training effect improved faster (**Figures 5A-C**). For the influence of different epochs on the model, the Accuracy-Loss curves showed that when the number of the epochs was increased, the fluctuation of the Accuracy curve and the Loss curve was similar (**Figures 5C-E**).

### Performance of the MDS Two-Classification Model

The performances of the MDS two-classification model with different outcome weights on the training set, testing set, and validation set were displayed in **Table 1**. When the outcome weight of the model was 1:9, the model had the best performances in the testing set. The AUC, accuracy, sensitivity, specificity, PPV, and NPV of the model were 0.984 (95%CI, 0.978-0.990), 0.909 (95%CI, 0.889-0.929), 0.971 (95%, 0.950-0.992), 0.882 (95%CI, 0.856-0.909), 0.783 (95%, 0.736-0.829), and 0.986 (95%CI, 0.975-0.996), respectively. In the external validation set, this model also had the highest AUC (0.965; 95%CI, 0.947-0.983), accuracy (0.935; 95%CI, 0.916-0.954), specificity (0.967; 95%CI, 0.950-0.983), and PPV (0.924, 95%CI, 0.887-0.961). The receiver operator characteristic (ROC) curves of the model with different outcome weights were shown in **Supplementary Figure 1**.

**Table 1:** The performances of the MDS two-classification model with different outcome weights.

Models	Data set	Sensitivity (95%CI)	Specificity (95%CI)	PPV (95%CI)	NPV (95%CI)	AUC (95%CI)	Accuracy (95%CI)
30 epochs, 5:5 outcome weight	Training set	0.984 (0.974-0.994)	0.905 (0.888-0.921)	0.818 (0.789-0.847)	0.992 (0.987-0.997)	0.982 (0.977-0.987)	0.929(0.917-0.940)
	Testing set	0.963 (0.939-0.987)	0.870 (0.842-0.898)	0.763 (0.715-0.811)	0.982 (0.970-0.994)	0.969 (0.959-0.978)	0.898(0.877-0.919)
	Validate set	0.852 (0.804-0.900)	0.934 (0.911-0.957)	0.856 (0.809-0.904)	0.932 (0.908-0.955)	0.931 (0.903-0.959)	0.908(0.886-0.930)
30 epochs, 2:8 outcome weight	Training set	0.989 (0.981-0.998)	0.898 (0.881-0.914)	0.808 (0.779-0.838)	0.995 (0.991-0.999)	0.985 (0.981-0.989)	0.925(0.913-0.937)
	Testing set	0.959 (0.933-0.984)	0.864 (0.836-0.893)	0.755 (0.707-0.803)	0.980 (0.967-0.992)	0.979 (0.972-0.986)	0.893(0.781-0.914)
	Validate set	0.886 (0.843-0.929)	0.934 (0.911-0.957)	0.861(0.815-0.907)	0.946 (0.925-0.967)	0.916 (0.886-0.945)	0.918(0.898-0.939)
30 epochs, 1:9 outcome weight	Training set	1.000 (1.000-1.000)	0.902 (0.886-0.918)	0.817 (0.788-0.846)	1.000 (1.000-1.000)	0.989 (0.986-0.992)	0.932(0.920-0.943)
	Testing set	0.971 (0.950-0.992)	0.882 (0.856-0.909)	0.783 (0.736-0.829)	0.986 (0.975-0.996)	0.984 (0.978-0.990)	0.909(0.889-0.929)
	Validate set	0.867 (0.821-0.913)	0.967 (0.950-0.983)	0.924 (0.887-0.961)	0.940 (0.918-0.961)	0.965 (0.947-0.983)	0.935(0.916-0.954)

CI, confidence interval; PPV, positive predictive value; NPV, negative predictive value; AUC, area under the curve.

When the outcome weight of the model was fixed at 1:9, and different epochs were used to train the model. The performances of the MDS two-classification model with different epochs on the training set, testing set, and validation set were shown in **Table 2**. The results indicated that both 30 epoch and 200 epoch models had good performance in the testing set. The AUC of the 30 epoch and 200 epoch models was 0.984 (95%CI, 0.978-0.990) and 0.985 (95%CI, 0.979-0.991), respectively. In the validation set, the 30 epoch and 200 epoch models still maintained good performance. The ROC curves of the model with different epochs were displayed in **Supplementary Figure 2**.

**Table 2:** The performances of the MDS two-classification model with different epochs.

Models	Data set	Sensitivity (95%CI)	Specificity (95%CI)	PPV (95%CI)	NPV (95%CI)	AUC (95%CI)	Accuracy (95%CI)
30 epochs, 1:9 outcome weight	Training set	1.000 (1.000-1.000)	0.902 (0.886-0.918)	0.817 (0.788-0.846)	1.000 (1.000-1.000)	0.989 (0.986-0.992)	0.932 (0.920-0.943)
	Testing set	0.971 (0.950-0.992)	0.882 (0.856-0.909)	0.783 (0.736-0.829)	0.986 (0.975-0.996)	0.984 (0.978-0.990)	0.909 (0.889-0.929)
	Validate set	0.867 (0.821-0.913)	0.967 (0.950-0.983)	0.924 (0.887-0.961)	0.940 (0.918-0.961)	0.965 (0.947-0.983)	0.935 (0.916-0.954)
50 epochs, 1:9 outcome weight	Training set	0.973 (0.960-0.987)	0.894 (0.878-0.911)	0.801 (0.771-0.831)	0.987 (0.981-0.994)	0.983 (0.979-0.988)	0.918 (0.906-0.931)
	Testing set	0.950 (0.923-0.978)	0.870 (0.842-0.898)	0.761 (0.713-0.809)	0.976 (0.962-0.989)	0.975 (0.967-0.983)	0.894 (0.873-0.916)
	Validate set	0.857 (0.810-0.904)	0.867 (0.836-0.899)	0.750 (0.695-0.805)	0.929 (0.904-0.953)	0.888 (0.855-0.921)	0.864 (0.838-0.890)
200 epochs, 1:9 outcome weight	Training set	0.998 (0.995-1.000)	0.907 (0.891-0.923)	0.824 (0.795-0.853)	0.999 (0.997-1.000)	0.991 (0.988-0.993)	0.935 (0.923-0.946)
	Testing set	0.983 (0.967-1.000)	0.875 (0.848-0.903)	0.775 (0.728-0.821)	0.992 (0.984-1.000)	0.985 (0.979-0.991)	0.908 (0.888-0.928)
	Validate set	0.895 (0.854-0.937)	0.942 (0.921-0.964)	0.879 (0.835-0.922)	0.951 (0.931-0.971)	0.924 (0.894-0.953)	0.927 (0.908-0.947)

CI, confidence interval; PPV, positive predictive value; NPV, negative predictive value; AUC, area under the curve.

### Performance of the AA, MDS, and AML Three-Classification Model

Similarly, **Table 3** demonstrates the performances of the AA, MDS, and AML three-classification model with different outcome weights on the training set, testing set, and validation set. Among the models with different outcome weights, the best model performance in the testing set was observed in the model with outcome weights of 1:9. The AUC, accuracy, sensitivity, specificity, PPV, and NPV of the model were 0.958 (95%CI, 0.948-0.968), 0.926 (95%CI, 0.913-0.939), 0.841 (95%CI, 0.810-0.871), 0.972 (95%CI, 0.962-0.982), 0.941 (95%CI, 0.921-0.962), and 0.920 (95%CI, 0.903-0.936), respectively. This model still had good performance in the validation set, with an AUC of 0.925 (95%CI, 0.909-0.941). The ROC curves of the model with different outcome weights were shown in **Supplementary Figure 3**.

**Table 3:** The performances of the AA, MDS, and AML three-classification model with different outcome weights.

Models	Data set	Sensitivity (95%CI)	Specificity (95%CI)	PPV (95%CI)	NPV (95%CI)	AUC (95%CI)	Accuracy (95%CI)
30 epochs, 5:5 outcome weight	Training set	0.884 (0.867-0.902)	0.960 (0.952-0.968)	0.922 (0.907-0.937)	0.940 (0.930-0.949)	0.970 (0.965-0.976)	0.934 (0.926-0.942)
	Testing set	0.834 (0.803-0.865)	0.952 (0.939-0.965)	0.902 (0.876-0.928)	0.915 (0.898-0.931)	0.945 (0.934-0.957)	0.911 (0.897-0.925)
	Validate set	0.858 (0.826-0.891)	0.880 (0.858-0.901)	0.787 (0.751-0.823)	0.923 (0.905-0.941)	0.911 (0.892-0.929)	0.872 (0.854-0.890)
30 epochs, 2:8 outcome weight	Training set	0.855 (0.836-0.874)	0.952 (0.943-0.960)	0.905 (0.888-0.921)	0.925 (0.914-0.935)	0.971 (0.966-0.976)	0.918 (0.909-0.927)
	Testing set	0.807 (0.774-0.839)	0.929 (0.913-0.944)	0.858 (0.828-0.888)	0.900 (0.882-0.918)	0.945 (0.933-0.956)	0.886 (0.870-0.902)
	Validate set	0.823 (0.788-0.858)	0.889 (0.868-0.910)	0.793 (0.757-0.830)	0.906 (0.887-0.926)	0.905 (0.886-0.924)	0.866 (0.848-0.885)
30 epochs, 1:9 outcome weight	Training set	0.890 (0.873-0.907)	0.986 (0.981-0.990)	0.970 (0.961-0.980)	0.944 (0.935-0.953)	0.976 (0.971-0.981)	0.952 (0.945-0.959)
	Testing set	0.841 (0.810-0.871)	0.972 (0.962-0.982)	0.941 (0.921-0.962)	0.920 (0.903-0.936)	0.958 (0.948-0.968)	0.926 (0.913-0.939)
	Validate set	0.852 (0.819-0.885)	0.901 (0.882-0.921)	0.817 (0.783-0.852)	0.921 (0.903-0.940)	0.925 (0.909-0.941)	0.884 (0.867-0.902)

CI, confidence interval; PPV, positive predictive value; NPV, negative predictive value; AUC, area under the curve.

The outcome weight of the model was chosen as 1:9 to compare the effects of different epochs on the three-classification model performance (**Table 4**). Compared with the 30 epoch and 50 epoch models, the 200 epoch model had the highest AUC (0.968; 95%CI, 0.960-0.976), accuracy (0.929; 95%CI, 0.916-0.941), sensitivity (0.857; 95%CI, 0.828-0.886), and NPV (0.927; 95%CI, 0.911-0.942). In addition, the performance of the 200 epoch model in the validation set was better than the 30 epoch and 50 epoch models. The ROC curves of the model with different epochs were demonstrated in **Supplementary Figure 4**.

**Table 4:** The performances of the AA, MDS, and AML three-classification model with different epochs.

Models	Data set	Sensitivity (95%CI)	Specificity (95%CI)	PPV (95%CI)	NPV (95%CI)	AUC (95%CI)	Accuracy (95%CI)
30 epochs, 1:9 outcome weight	Training set	0.890 (0.873-0.907)	0.986 (0.981-0.990)	0.970 (0.961-0.980)	0.944 (0.935-0.953)	0.976 (0.971-0.981)	0.952 (0.945-0.959)
	Testing set	0.841 (0.810-0.871)	0.972 (0.962-0.982)	0.941 (0.921-0.962)	0.920 (0.903-0.936)	0.958 (0.948-0.968)	0.926 (0.913-0.939)
	Validate set	0.852 (0.819-0.885)	0.901 (0.882-0.921)	0.817 (0.783-0.852)	0.921 (0.903-0.940)	0.925 (0.909-0.941)	0.884 (0.867-0.902)
50 epochs, 1:9 outcome weight	Training set	0.892 (0.875-0.909)	0.963 (0.956-0.971)	0.928 (0.914-0.942)	0.944 (0.934-0.953)	0.975 (0.970-0.979)	0.938 (0.931-0.946)
	Testing set	0.850 (0.820-0.880)	0.958 (0.946-0.971)	0.916 (0.892-0.940)	0.923 (0.907-0.939)	0.951 (0.939-0.963)	0.921 (0.907-0.934)
	Validate set	0.834 (0.800-0.868)	0.912 (0.893-0.931)	0.830 (0.796-0.865)	0.914 (0.895-0.932)	0.894 (0.873-0.916)	0.885 (0.868-0.902)
200 epochs, 1:9 outcome weight	Training set	0.901 (0.884-0.917)	0.983 (0.977-0.988)	0.965 (0.955-0.975)	0.949 (0.940-0.957)	0.983 (0.979-0.987)	0.954 (0.947-0.961)
	Testing set	0.857 (0.828-0.886)	0.967 (0.956-0.978)	0.933 (0.911-0.955)	0.927 (0.911-0.942)	0.968 (0.960-0.976)	0.929 (0.916-0.941)
	Validate set	0.887 (0.858-0.916)	0.929 (0.912-0.946)	0.866 (0.835-0.897)	0.941 (0.925-0.957)	0.948 (0.935-0.961)	0.915 (0.900-0.930)

CI, confidence interval; PPV, positive predictive value; NPV, negative predictive value; AUC, area under the curve.

### Final Recognition Model

According to the evaluation indicators of the model in the results of the two-classifications and three-classification, when the epoch of the model was 200 and the outcome weight was 1:9, the model had better performance in the two-classification of MDS and the three-classification of AA, MDS, and AML. Therefore, the epoch was 200 and the outcome weight was 1:9 as the final model used. The performances of the final model were shown in **Table 5**. The ROC curves of the final model were demonstrated in **Figure 6**. In the final model construction, each epoch took 4 minutes, the model took 800 minutes on the training set and 5 minutes on the testing set. When the model is used in practice, the result can be obtained in 0.3 seconds after inputting a single bone marrow image of patients.

**Table 5:** The performances of the final two-classification and the three-classification models.

Models	Data set	Sensitivity (95%CI)	Specificity (95%CI)	PPV (95%CI)	NPV (95%CI)	AUC (95%CI)	Accuracy (95%CI)
Two-classification (200 epochs, 1:9)	Training set	0.996 (0.992-1.000)	0.911 (0.895-0.926)	0.830 (0.802-0.858)	0.998 (0.996-1.000)	0.991 (0.988-0.993)	0.937 (0.926-0.948)
	Testing set	0.992 (0.980-1.000)	0.881 (0.854-0.908)	0.784 (0.737-0.830)	0.996 (0.990-1.000)	0.985 (0.979-0.991)	0.914 (0.895-0.934)
	Validate set	0.886 (0.843-0.929)	0.938 (0.916-0.960)	0.869 (0.824-0.914)	0.946 (0.926-0.967)	0.942 (0.918-0.967)	0.921 (0.901-0.942)
Three-classification (200 epochs, 1:9)	Training set	0.901 (0.884-0.917)	0.983 (0.977-0.988)	0.965 (0.955-0.975)	0.949 (0.940-0.957)	0.983 (0.979-0.987)	0.954 (0.947-0.961)
	Testing set	0.857 (0.828-0.886)	0.967 (0.956-0.978)	0.933 (0.911-0.955)	0.927 (0.911-0.942)	0.968 (0.960-0.976)	0.929 (0.916-0.941)
	Validate set	0.887 (0.858-0.916)	0.929 (0.912-0.946)	0.866 (0.835-0.897)	0.941 (0.925-0.957)	0.948 (0.935-0.961)	0.915 (0.900-0.930)

CI, confidence interval; PPV, positive predictive value; NPV, negative predictive value; AUC, area under the curve.

## Discussion

In this study, a recognition model based on bone marrow smears was constructed using deep learning to distinguish whether the patient was MDS and which of AA, MDS, and AML the patient was. The AUC and accuracy of the model to classify patients as MDS were 0.985 and 0.914, respectively. When the model was used to distinguish AA, MDS, and AML, the AUC and accuracy of the model were 0.968 and 0.929, respectively. In addition, the model still retained a good distinguishing ability in external validation.

It has been reported that the risk of MDS/AML in AA patients increased with the duration of the disease without reaching a plateau, occurring in 4%-8% of patients at 5-6 years of follow-up and in 9%-26% of patients at 10 years (23, 24). Among MDS patients, approximately 30% will experience the evolution of AML (25). Furthermore, patients with MDS are at a much greater risk of progressing to AML than those with AA (26). Therefore, the distinction between MDS and AA is very important in disease control and treatment. The diagnosis of these diseases depends on the judgment of the clinicians on the diagnostic tests (17). Deep learning can assist clinicians in the recognition of image results. The deep learning CNN method can imitate the natural visual processing in the brain and can interpret dense information (10). In clinical practice, the use of deep learning method to assist clinicians in processing the images of detection results may be able to avoid the impact of differences in experience between clinicians on the diagnosis results, and it has been applied to disease identification (27–29). For example, Shafique et al. utilized the deep learning CNN features for the typing of acute lymphoblastic leukemia cells, and the sensitivity and specificity of the model achieve 95%-99% (28).

A deep learning model for identifying MDS patients by dysplastic neutrophils in peripheral blood was constructed by Acevedo et al. and the model achieved 95.5% sensitivity and 94% accuracy (30). The sensitivity and accuracy of our bone marrow smear-based deep learning model for identifying MDS patients were 99.2% and 91.4%, respectively. However, few studies have reported models based on deep learning to distinguish AA, MDS, and AML. Only a recent study conducted by Kimura et al. used the deep learning method to distinguish AA and MDS (14). Their CNN model was based on peripheral blood indicators to identify MDS from AA patients, and the AUC and sensitivity of the model were 0.990 and 0.962, respectively. However, their model used many blood indicators, which may not be convenient in clinical practice, and the model lacked external validation. In the current study, we also constructed a CNN model only based on bone marrow smears to distinguish AA, MDS, and AML. The model demonstrated a good ability to distinguish AA, MDS, and AML, and the AUC of the model in the testing set and external validation set were 0.968 and 0.948, respectively. Our model was validated by external clinical data, and the results showed that the model was reliable in clinical practice. In addition, our model was more convenient in clinical practice. By inputting the patient’s bone marrow smear image into our model, after 0.3 seconds we can know whether the patient has MDS, or which of the patient has AA, MDS, and AML. Our model may provide clinicians with a convenient and effective tool to distinguish AA, MDS, and AML. The use of deep learning for disease recognition is to extract relevant features based on the identified disease diagnosis images. Therefore, the wider application of deep learning to disease recognition depends on more manual diagnosis results. In addition to disease identification, future studies may need to focus on the related disease progression, such as predicting the risk of AA and MDS progression to AML through deep learning methods.

Our study has constructed a model that can identify whether a patient has MDS, and can distinguish which of the AA, MDS, and AML diseases the patient has. In addition, our model has been validated by external clinical data to ensure the applicability of the model in clinical practice, and the model had a good ability to distinguish AA, MDS, and AML. However, some limitations of this study should be considered. First, the sample size of our study was relatively small, and larger sample size studies may be needed in the future. Second, although we used data enhancement methods to increase the samples, there were still differences between the increased sample and the independent individual sample, which may have an impact on our results. Third, we did not analyze the different subtypes of MDS and AML due to the limited sample size of the ASH Image Bank. Fourth, we cannot identify the features of cases that are discordant or misclassified with the model due to the features extracted by deep learning in a single image are unknown. Firth, the differences between database patients and hospital patients could not be analyzed due to the lack of relevant characteristics of patients in the database, which may affect the extrapolation of our results. However, the good performance of the model on the external validation set indicates that the model is robust.

## Conclusions

The image-net pretrained model had high recognition accuracy in the two-classification of MDS and the three-classification of AA, MDS, AML. This model can help clinicians identify whether the patient had MDS through the patient’s bone marrow smear image, and distinguish the types of AA, MDS, and AML. This model may provide clinicians with a convenient tool to distinguish AA, MDS, and AML in clinical practice.

## Data Availability Statement

The raw data supporting the conclusions of this article will be made available by the authors, without undue reservation.

## Ethics Statement

The studies involving human participants were reviewed and approved by The Second Hospital of Shanxi Medical University. The patients/participants provided their written informed consent to participate in this study.

## Author Contributions

MW designed the study and wrote the manuscript. CD, YG, JL, MH and LW collected, analyzed and interpreted the data. MW critically reviewed, edited and approved the manuscript. All authors read and approved the final manuscript.

## Funding

This work was supported by the Key Research and Development Projects of Shanxi Province (No. 201803D31123).

## Conflict of Interest

The authors declare that the research was conducted in the absence of any commercial or financial relationships that could be construed as a potential conflict of interest.

## Publisher’s Note

All claims expressed in this article are solely those of the authors and do not necessarily represent those of their affiliated organizations, or those of the publisher, the editors and the reviewers. Any product that may be evaluated in this article, or claim that may be made by its manufacturer, is not guaranteed or endorsed by the publisher.

## Supplementary Material

Supplementary Figure 1 ROC curves of the two-classification model with different outcome weights. **(A)** ROC curves of 5:5 outcome weight; **(B)** ROC curves of 2:8 outcome weight; **(C)** ROC curves of 1:9 outcome weight.

Supplementary Figure 2 ROC curves of the two-classification model with different epochs. **(A)** ROC curves of 30 epochs; **(B)** ROC curves of 50 epochs; **(C)** ROC curves of 200 epochs.

Supplementary Figure 3 ROC curves of the three-classification model with different outcome weights. **(A)** ROC curves of 5:5 outcome weight; **(B)** ROC curves of 2:8 outcome weight; **(C)** ROC curves of 1:9 outcome weight.

Supplementary Figure 4 ROC curves of the three-classification model with different epochs. **(A)** ROC curves of 30 epochs; **(B)** ROC curves of 50 epochs; **(C)** ROC curves of 200 epochs.
