# Supplementary figures and images for: A Deep Learning Model for the Automatic Recognition of Aplastic Anemia, Myelodysplastic Syndromes, and Acute Myeloid Leukemia Based on Bone Marrow Smear
Source: Front Oncol. 2022 Apr 14;12:844978. doi: 10.3389/fonc.2022.844978 (PMC9047549; doi:10.3389/fonc.2022.844978)

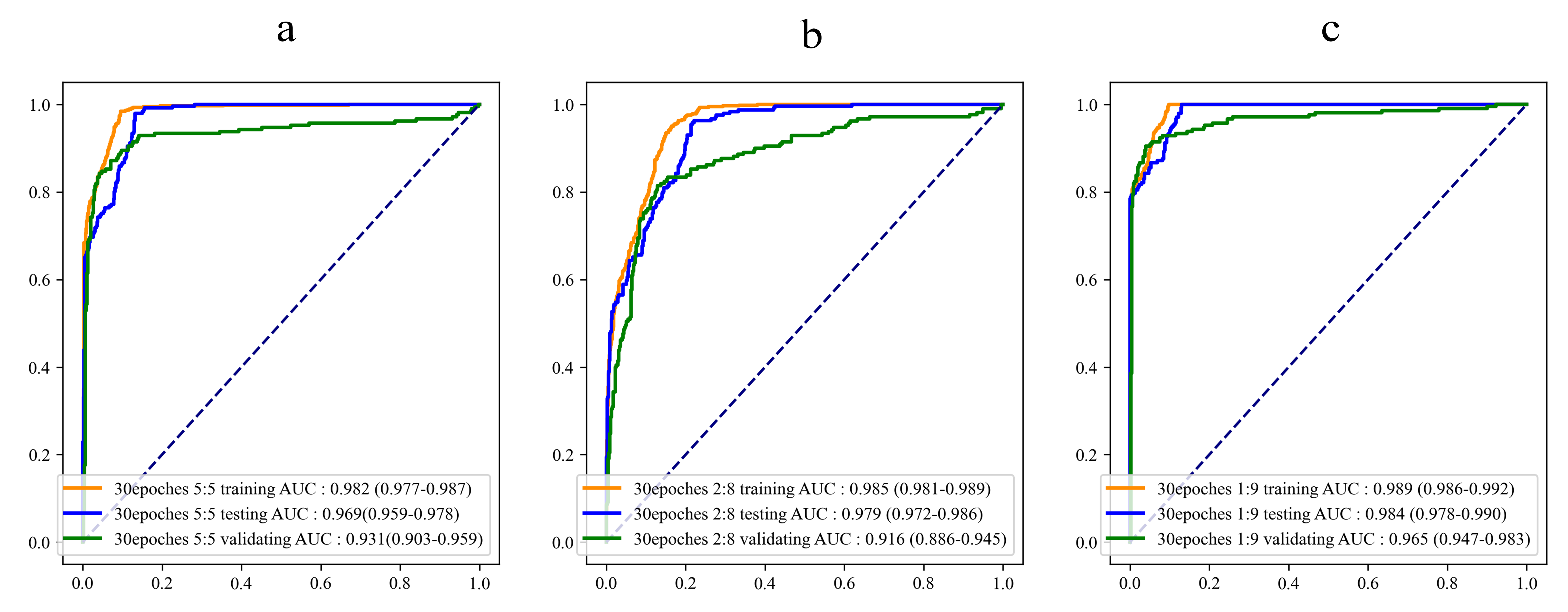

Supplement: Supplementary Figure 1 — ROC curves of the two-classification model with different outcome weights. (A) ROC curves of 5:5 outcome weight; (B) ROC curves of 2:8 outcome weight; (C) ROC curves of 1:9 outcome weight. [file Image_1.tif]

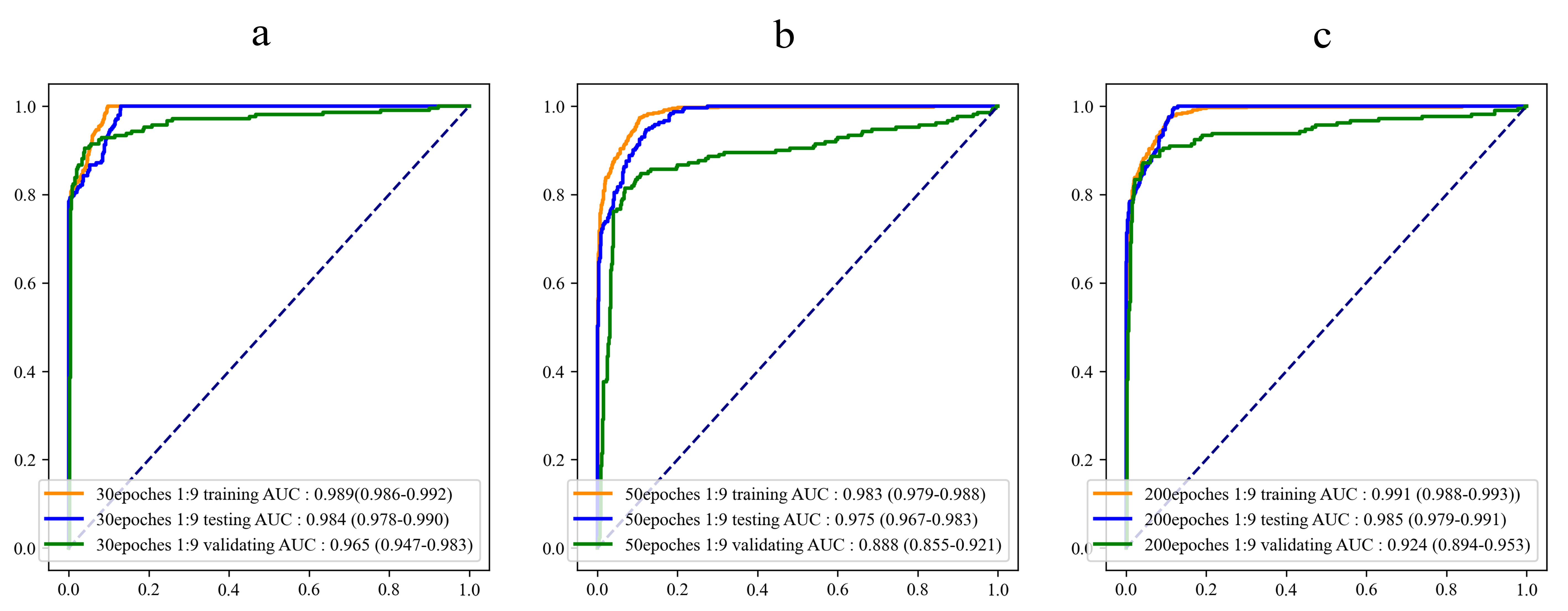

Supplement: Supplementary Figure 2 — ROC curves of the two-classification model with different epochs. (A) ROC curves of 30 epochs; (B) ROC curves of 50 epochs; (C) ROC curves of 200 epochs. [file Image_2.tif]

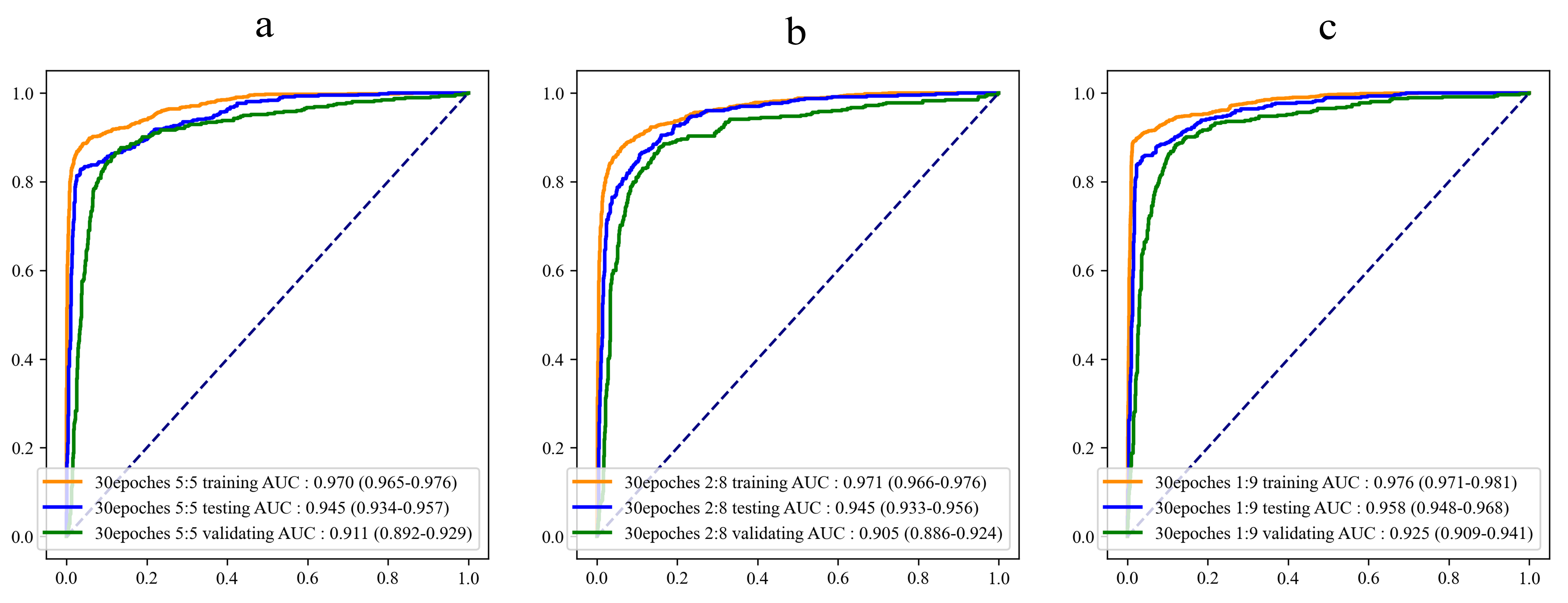

Supplement: Supplementary Figure 3 — ROC curves of the three-classification model with different outcome weights. (A) ROC curves of 5:5 outcome weight; (B) ROC curves of 2:8 outcome weight; (C) ROC curves of 1:9 outcome weight. [file Image_3.tif]

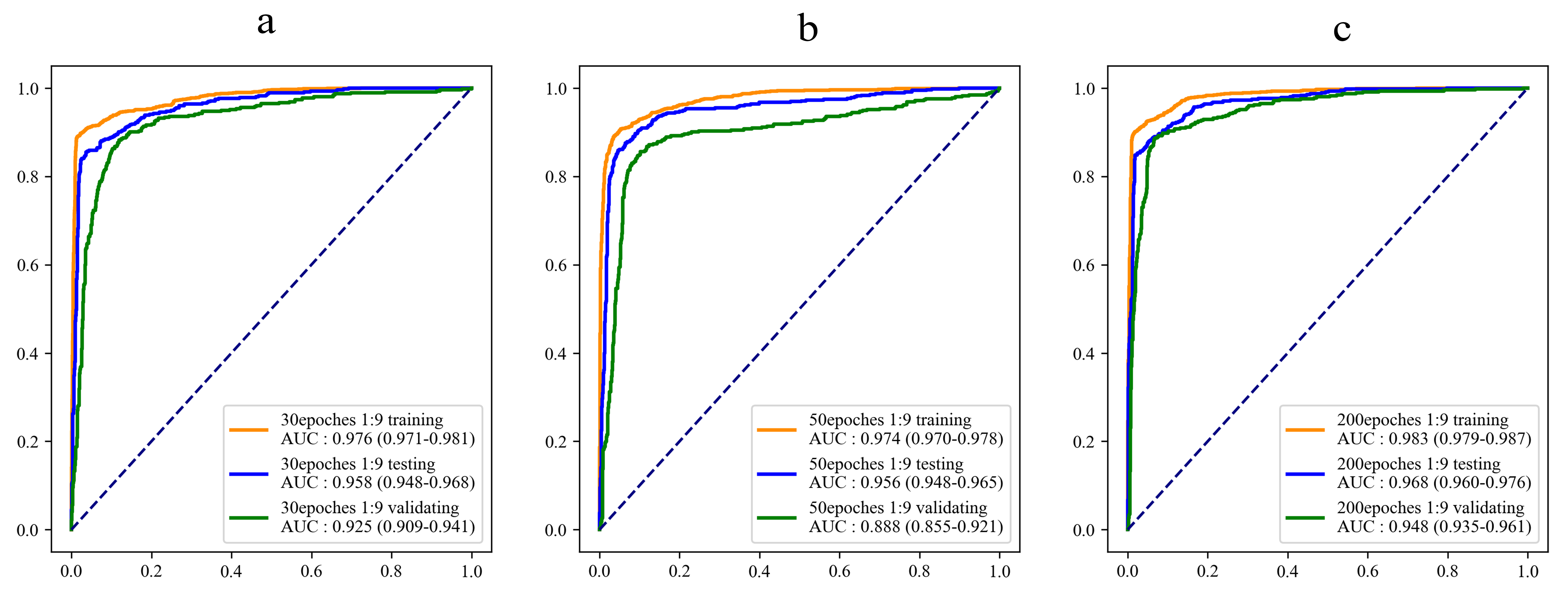

Supplement: Supplementary Figure 4 — ROC curves of the three-classification model with different epochs. (A) ROC curves of 30 epochs; (B) ROC curves of 50 epochs; (C) ROC curves of 200 epochs. [file Image_4.tif]
